# The evolving role of long noncoding RNA HIF1A-AS2 in diabetic retinopathy: a cross-link axis between hypoxia, oxidative stress and angiogenesis via MAPK/VEGF-dependent pathway

**DOI:** 10.1080/13510002.2022.2050086

**Published:** 2022-03-14

**Authors:** Marwa Mohamed Atef, Noha M. Shafik, Yasser Mostafa Hafez, Mona Mohamed Watany, Amal Selim, Heba M. Shafik, Omnia Safwat El-Deeb

**Affiliations:** a Medical Biochemistry Department, Faculty of Medicine, Tanta University, Tanta, Egypt; b Internal Medicine Department, Faculty of Medicine, Tanta University, Tanta, Egypt; c Clinical pathology Department, Faculty of Medicine, Tanta University, Tanta, Egypt; d Ophthalmology Department, Faculty of Medicine, Tanta University, Tanta, Egypt

**Keywords:** Diabetic retinopathy, HIF1A-AS2, HIF-1α, VEGF, MAPK, endoglin, oxidative stress, angiogenesis

## Abstract

**Background:**

Diabetic retinopathy (DR) signifies a frequent serious diabetic complication influencing retinal structure and function. Dysregulation of lncRNAs drives a wide array of human diseases especially diabetes; thus, we aimed to study lncRNA HIF1A-AS2 role and its interplay with hypoxia, oxidative stress (OS), and angiogenesis in DR.

**Materials and methods:**

60 DM patients in addition to 15 healthy subjects. were enrolled. LncRNA HIF1A-AS2 mRNA relative gene expression was assessed. Hypoxia inducible factor 1-alpha (HIF-1α), vascular endothelial growth factor (VEGF), mitogen activated protein kinase (MAPK), and endoglin levels were assessed. Detection of DNA damage using comet assay, and Redox status parameters were also detected.

**Results:**

LncRNA HIF1A-AS2 expression was significantly increased in diabetic patients with the highest levels in proliferative DR patients. Moreover, HIFα, VEGF, MAPK, and Endogolin levels were significantly higher in the diabetic patients compared to control group with the highest levels in in proliferative DR patients. Significant DNA damage in comet assay was observed to be the highest in this group.

**Conclusion:**

We observed for the first time the imminent role of long noncoding RNA HIF1A-AS2 in DR throughout its stages and its interplay with hypoxia, OS, and angiogenesis via MAPK/VEGF-dependent pathway.

## 1. Introduction

Diabetic retinopathy (DR) is identified to be one of the most communal causes of visual impairment worldwide as it is the principal cause of blindness in adults aged between 27 and 75, and by 2030, 191 million people are expected to be diagnosed with this microvascular condition [1]. DR is categorized into nonproliferative diabetic retinopathy (NPDR) and proliferative diabetic retinopathy (PDR) according to the existence of noticeable ophthalmological alterations and retinal neovascularization [2]. NPDR is commonly symptomless except when linked to macular edema; nevertheless, patients with un-controlled DM or when retinopathy’s advancement is not observed tend to develop PDR [3].

Almost eighty percent of the human genome is transcribed in a cell-specified way, predominantly noncoding regions [4]. Only a minor portion of it is transcribed into protein-coding mRNAs, and the massive common ones produces plentiful long noncoding RNAs (lncRNAs) [5] that comprise numerous RNA species longer than 200 nucleotides that are not translated into proteins [6].

An lncRNA can be positioned into one or more of five comprehensive subcategories: sense, antisense, intronic, intergenic, and bidirectional. Emerging evidences suggest that lncRNAs have been recently revealed to play imperative regulatory roles in fundamental biological processes, and voluminous numbers of them are deregulated in several human diseases [7]. Although poorly described, it is documented that they are capable of accomplishing numerous tasks such as post-transcriptional regulation, cell–cell signaling, organization of protein complexes, and their allosteric regulation [8].

HIF1A-AS2 is an antisense lncRNA, which is an antisense transcript of hypoxia-inducible factor 1alpha (HIF-1α) [9]. It was explicitly overexpressed in many diseases as nonpapillary clear cell renal carcinomas [10]. Formerly, it was revealed that it could be detected in normal human tissues in both fetal and adulthood periods as in tumor tissues [11].

As with other lncRNAs, HIF1A-AS2 plays a dynamic role in cancer development, through adapting the cancer-related HIF-1α pathway [12]. It was also documented to be involved in metabolic processes regulation as it enterprises a positive feedback loop of glycolysis and HIF-1α arbitrated trans-activation [13].

Acute or lingering exposure to the diabetic milieu results in a diversity of metabolic and biochemical aberrations, which while interrelated may be also concurrently triggered. Considerable research work introduced various mechanisms for its occurrence comprising polyol pathway [14], advanced glycation end products (AGEs) pathway [15], MAPK pathway, haemodynamic alterations in blood flow [16], angiogenic pathway [17], as well as pathways associated with oxidative damage [18]. Though the exact pathogenesis of DR remains partly understood, oxidative stress (OS), hypoxia associated processes, and inflammation are supposed to contribute toward DR development [19].

Body of confirmation has revealed that vascular endothelial growth factor (VEGF) plays a substantial role in the angiogenesis and proliferation of DR and that its transcriptional regulation is partially arbitrated by HIF-1α that is involved in apoptosis, hypoxic adaptation, and neovascularization [20]. Under standard conditions, the HIF-1α protein is unstable and degrades quickly, making it hard to be identified in retinal tissue [21]. However, under hypoxic condition, there is a considerable escalation in HIF-1α levels. When overexpressed, HIF-1α and its down-stream regulatory factors cause retinal neovascularization, which contribute to PDR occurrence [22].

**Aim of the work:** Given the significance of the main role played by lncRNA in variable diseases, the study was done to clarify the biological machineries by which HIF1A-AS2 acts in DR pathogenesis being linked to hypoxic conditions, OS, and DNA damage.

## 2. Subjects and methods

### 2.1. Study population

A cross-sectional study of 60 subjects attended to Tanta University Hospitals who were diagnosed to have diabetes mellitus type 2 according to the American Diabetes Association criteria [23] whether suffering of visual complaints or accidentally discovered retinal affection by routine investigations before the occurrence of any visual complains with various durations of illness and different degrees of glycemic control. Full ophthalmological evaluation of the patients for visual acuity, refraction, anterior segment examination by slit lamp, ND fundus examination by fluorescein angiography was done for all patients by author 6 at Ophthalmology Department, Tanta University Hospitals. The patients were categorized into three groups using fundus fluorescein angiography examination in accordance to the designated staging criteria in the fundi disease academic conference in 2002 [24]: 15 type 2 diabetes mellitus (T2DM) patients who did not suffer from any signs of diabetic retinopathy, 15 T2DM patients with nonproliferative diabetic retinopathy, and 15 T2DM patients with proliferative diabetic retinopathy. In addition, 15 age- and gender-matched healthy subjects who were not diagnosed to have diabetes were taken as a control group**.**

The study design was approved from Research Ethics Committee guidelines, Faculty of Medicine, Tanta University and was in agreement with the principles of Declaration of Helsinki II. All participants sign an informed consent before being enrolled.

***Exclusion criteria:***

- Previous intraocular surgery (except cataract surgery done more than 6 months before the study).
- Coincident retinal pathology, choroidal neovascular membrane, and age-related macular degeneration.
- Prior ocular inflammation.
- Renal impairment, cardiovascular disease, malignancy, and autoimmune or inflammatory disorders.

### 2.2. Methods

All subjects were subjected to detailed clinical history, experienced ophthalmologic and physical examination included anthropometric measurements.

#### 2.2.1. Blood sampling

Blood samples were collected following sterile procedures and divided into three parts: one part was collected into a dry sterile centrifuge tube, left to clot, centrifuged at 1,200 × g for 15 min, and serum was collected for biochemical analysis, and the second part was collected in ethylenediaminetetraacetic acid (EDTA)-treated tubes for percentage estimation of HbA1c using colorimetric technique as total hemoglobin percentage and for advanced glycation end products (AGEs) spectrofluorometric assessment [25]. The last part was collected in heparinized tubes and used for preparation of peripheral blood mononuclear cells (PBMCs). All samples were saved frozen after collection at −80 °C till analysis.

Early morning single-spot urine samples were collected from all participants for estimation of urinary albumin/creatinine ratio (UACR), the samples were immediately centrifuged, and urinary creatinine was measured using colorimetric kit obtained from (Diamond Diagnostics, Egypt). And, urinary albumin was estimated by immunoturbidimetric method (BioSystems, Spain), and lastly, UACR (mg/g) was estimated.

#### 2.2.2. Preparation of peripheral blood mononuclear cells (PBMCs)

PBMCs were prepared using Ficoll-Hypaque (Pharmacia, Uppsala, Sweden) via density gradient centrifugation procedure [26]. Heparinized blood was cautiously layered on Ficoll, after a centrifugation with Ficoll-Hypaque; platelets and plasma are located above the Ficoll-Hypaque, while lymphocytes and some platelets are found at the plasma–Ficoll-Hypaque interface. While at the bottom of the tube, a cell pellet made of granulocytes and RBCs is obtained. Then, PBMCs were harvested from the white interphase between the plasma and the Ficoll-Hypaque layers after performing centrifugation at 400 g at room temperature for a period of 30 min, washed using phosphate buffered saline, and finally, PBMCs samples were frozen at −80°C and used later for RNA isolation and comet assay.

#### 2.2.3. Biochemical analysis

**1.** *Assessment of serum hypoxia inducible factor 1-alpha (HIF-1α) and vascular endothelial growth factor (VEGF) levels* using ELISA kits purchased from (MyBioSource, San Diego, CA, USA), following the manufacturer’s protocol using ELISA Reader (Stat Fax®2100, Fisher Bioblock Scientific, France).
**2.** *Assessment of serum mitogen activated protein kinase (MAPK) and endoglin levels* using ELISA kits obtained from (MyBioSource, San Diego, CA, USA), according to the manufacturer’s steps.
**3.** Assessment of HIF1A antisense RNA 2 (HIF1A-AS2) expression level by quantitative real-time PCR
3.1. Total RNA was isolated from PBMC samples by Qiagen RNeasy Mini Kit supplied by Qiagen (Valencia, CA, USA) as supposed by the manufacturer protocol. Assessment of RNA concentration and purity using NanoDrop spectrophotometer (NanoDrop Technologies, Inc. Wilmington, NC, USA) through measurement of OD260 and OD260/280 ratio, respectively. RNA was then frozen at −80°C.
3.2. Then reverse transcription of the extracted RNA into cDNA was performed using high-capacity cDNA synthesis kit Transcription kit purchased from (Applied Biosystem, San Francisco, CA, USA) according to the manufacturer’s manuals.
3.3. Real-time PCR using a StepOnePlus Real-Time PCR system (Applied Biosystem, San Francisco, CA, USA) was used to detect HIF1A–AS2 gene expression as follows: initial denaturation at 95°C for 10 min, followed by 40 cycles with denaturation at 95°C for 15 s, annealing at 60°C for 30 s, and extension at 72°C for 30 s. Primer sequences specific for HIF1A–AS2 were designed as follows: F, 5′-TCTGTGGCTC AGTTCCTTTTGT-3′ and R, 5′-ATGTAGGAAGTGCCAGAGCC-3′ with (NCBI GenBank Nucleotide accession # NR_045406). (Each run included a positive control and nontemplate control (negative control) to control the quality of PCR.) Primers for glyceraldehyde-3-phosphate dehydrogenase (GAPDH) that was used as an endogenous control have primer sequences of F, 5′-CTCGCTTCGGCAGCACA-3′, and R, 5′-AACGCTTCACGAATTTGCGT-3′ with (NCBI GenBank Nucleotide accession # NR_004394.1). Relative levels of gene expression were determined using 2^-ΔΔCt^ method [27] and normalized to the reference gene used (GAPDH).
**4.** Redox status markers:
4.1. **Serum malondialdehyde (MDA) level** was measured as stated by Ohkawa et al. [28] MDA level was calculated at 532 nm via an extinction coefficient of MDA–thiobarbituric acid complex, which is 1.56 × 10^5^ M^−1^ cm^−1^.
4.2. **Serum total antioxidant capacity (TAC) level** was assessed using commercial colorimetric kit supplied by (Biodiagnostic, Giza, Egypt) according to Koracevic et al. [29].
4.3. **Serum total thiols level** was assessed according to Hu method [30]. Spectrophotometric assessment of thiol groups concentrations was calculated at 412 nm using extinction coefficient of 13.600 M^−1^ cm^−1^.
**5.** Assessment of serum nitric oxide (NO) and peroxynitrite levels
5.1. **Serum nitric oxide (NO) level** was assessed using commercial colorimetric kit supplied by (Biodiagnostic, Giza, Egypt) according to Montgomery et al. [31].
5.2. **Serum peroxynitrite (ONOO^−^) level** was measured according to Beckman et al. [32] in which the peroxynitrite mediated nitration of phenol was measured spectrophotometrically at 412 nm. ONOO− level was calculated using extinction coefficient of 4400 M^−1^ cm^−1^.
**6.** *Comet assay*: Alkaline comet assay was evaluated on PBMCs from all participants using a commercial kit obtained from Trevigen’s Comet Assay kit (Gaithersburg, MD, USA) following kit instructions. DNA was stained with propidium iodide. Stained slides were evaluated using a 40× objective on a fluorescent microscope with excitation filter of 420–490 nm (Olympus BX51, Tokyo, Japan). Komet 5 image analysis software developed by Kinetic Imaging, Ltd. (Liverpoo1,UK) linked to a CCD camera was used for assessment of qualitative and quantitive DNA damage in the cells by evaluating the length of DNA migration, migrated DNA percentage, and tail moment. In general, 50–100 randomly selected cells are evaluated per sample [33].

### 2.3. Statistical evaluation

Statistical analysis was achieved using the computer SPSS program (Statistical Package for the Social Science; SPSS, Chicago, IL, USA) version 21 for Microsoft Windows, USA. Variables were presented as means ± SD. Statistical differences between variables were conducted using one-way analysis of variance (ANOVA) followed by post hoc analysis. The relationship between different parameters was performed using Pearson correlation. Multiple linear regression analysis was performed to assess the factors influencing HIF1A–AS2 expression with related factors as independent variables. Analysis was finally completed using sensitivity and specificity, and the best cutoff point was determined via Receiver Operating Characteristic (ROC) curve. The Kolmogorov–Smirnov (KS) test is used to test the null hypothesis that a set of data comes from a Normal distribution. *P* value of < 0.05 was considered statistically significant.

## 3. Results

### 3.1. Clinical characteristics

As presented in Table 1, no significant variance was found concerning age and gender. A statistically significant difference in DM duration between the diabetic groups was detected; the duration of DM was longer in PDR patients. A significant difference was found also in BMI. FBG, PpBG, and HbA1c were significantly higher in the three diabetic groups compared with healthy control with the highest values detected in the PDR group. A significant difference regarding lipid profile was also revealed between the studied groups. A significantly higher level of UACR was detected in T2DM patients in comparison with control groups, with highest values found in PDR patients.

**Table 1.** Demographic and clinical data of the studied groups.

Variable	Control	Diabetic without retinopathy	Nonproliferative diabetic retinopathy	Proliferative diabetic retinopathy	*P* value
Gender					
Male	8(53.33%)	7 (46.67%)	8(53.33%)	9(60.00%)	
Female	7 (46.67%)	8(53.33%)	7 (46.67%)	6(40.00%)	
Age (years)	50.47 + 8.32	52.53 + 9.91	50.67 + 10.66	54.02 + 9.13	**0.840**
Duration (years)	–	2.60 + 1.24 *^▴◊^	7.47 + 2.33*^# ◊^	13.60 + 5.41*^#▴^	<0.001*
BMI (kg/m^2^)	28.35 + 2.30	33.82 + 3.37*	35.12 + 3.54*	34.45 + 3.04*	<0.001*
FBG (mg/dl)	91.33 + 2.74	171.47 + 27.11*^▴◊^	205.80 + 27.69*^#^	225.20 + 40.87*^#^	<0.001*
Pp. BG (mg/dl)	115.27 + 12.03	228.53 + 39.78*^▴◊^	275.33 + 35.10*^#^	290.60 + 61.68*^#^	<0.001*
HbA1c (%)	4.98 + 0.23	7.5 + 0.70*^▴◊^	8.95 + 0.98 *^# ◊^	9.75 + 1.06*^#▴^	<0.001*
TC (mg/dl)	189.47 + 14.95	216.73 + 24.02^▴◊^	256.87 + 41.07*^#^	264.47 + 44.83*^#^	<0.001*
TG (mg/dl)	133.17 + 6.88	187.33 + 11.52*^▴◊^	233.27 + 29.93*^# ◊^	273.40 + 31.69*^#▴^	<0.001*
HDL-c (mg/dl)	46.67 + 2.38	42.60 + 3.52* ^◊^	41.73 + 4.48* ^◊^	39.33 + 3.38*^#▴^	<0.001*
LDL-c (mg/dl)	116.17 + 0.37	136.66 + 0.49*^▴◊^	168.49 + 0.52*^# ◊^	170.48 + 0.71*^#▴^	<0.001*
UACR (mg/g)	23.47 + 2.80	201.27 + 31.52*^▴◊^	286.47 + 35.33*^# ◊^	361.0 + 81.41*^#▴^	<0.001*

Abbreviation: BMI: body mass index; FBG: fasting blood glucose; Pp. BG: 2h-post prandial blood glucose; HbA1c: glycated hemoglobin; TC: total cholesterol; TG: triacylglycerol; HDL-c: high-density lipoprotein–cholesterol; LDL-c: low-density lipoprotein–cholesterol; UACR: urinary albumin-to-creatinine ratio.

Statistical study is achieved using one-way ANOVA with Tukey’s post hoc test, SPSS computer program.

* Significant difference vs. control group (*P* < 0.05).

# Significant difference vs. diabetic group without retinopathy (*P* < 0.05).

▴Significant difference vs. nonproliferative diabetic retinopathy group (*P* < 0.05).

◊ Significant difference vs. proliferative diabetic retinopathy group (*P* < 0.05).

### 3.2. Biochemical results

As presented in Table 2, the mean values of HIF-1α, VEGF, MAPK, Endoglin, and AGEs levels were significantly increased in the diabetic groups when compared with those stated in the control healthy group with significantly higher levels noted in PDR group. Redox status imbalance as evidenced by marked increase in MDA level with concomitant decrease in TAC, and total thiols levels were detected in diabetic groups especially in PDR group.

**Table 2.** Biochemical parameters of the studied groups.

Variable	Control	Diabetic without retinopathy	Nonproliferative diabetic retinopathy	Proliferative diabetic retinopathy	*P* value
HIF-1α level (pg/ml)	87.60 ± 9.53	226.40 ± 42.25*^▴◊^	393.99 ± 57.42*^# ◊^	712.65 ± 84.74*^#▴^	**<0**.001*
VEGF level (pg/ml)	98.51 ± 17.55	154.36 ± 22.40*^▴◊^	223.58 ± 24.33 *^#^	244.16 ± 40.87*^#^	**<0**.001*
Endoglin level (ng/ml)	0.58 ± 0.17	2.12 ± 0.43*^▴◊^	3.34 ± 0.45 *^# ◊^	4.69 ± 0.75 *^#▴^	**<0**.001*
MAPK level (pg/ml)	42.54 ± 6.62	69.18 ± 8.64*^▴◊^	85.58 ± 10.19*^# ◊^	99.40 ± 20.79*^#▴^	**<0**.001*
AGEs (U/ml)	34.60 ± 6.98	74.20 ± 9.99*^▴◊^	93.60 ± 10.53*^# ◊^	119.10 ± 12.81*^#▴^	**<0**.001*
MDA level (nmol/L)	61.87 ± 9.46	97.51 ± 10.49*^▴◊^	126.05 ± 11.56*^# ◊^	173.44 ± 24.43*^#▴^	**<0**.001*
TAC level (mmol/L)	1.65 ± 0.15	1.03 ± 0.21*^▴◊^	0.91 ± 0.23*^# ◊^	0.87 ± 0.36*^#▴^	**<0**.001*
Total thiols level (μmol/ml)	271.41± 12.60	219.57 ± 14.70*^▴◊^	192.28 ± 13.35*^# ◊^	173.39 ± 29.88*^#▴^	**<0**.001*
NO level (µmol/L)	31.77 ± 5.97	44.15± 4.28*^▴◊^	51.52 ± 5.35*^# ◊^	59.11 ± 9.84*^#▴^	**<0**.001*
ONOO^−^ level (µmol/L)	3.91 ± 0.41	6.63 ± 0.55*^▴◊^	8.24 ± 0.61*^#^	8.92 ± 1.67 *^#^	**<0**.001*

Abbreviation: AGEs: advanced glycation end products; HIF-1α: hypoxia inducible factor 1-alpha; MAPK: mitogen activated protein kinase; MDA: malondialdehyde; NO: nitric oxide; ONOO−: peroxynitrite; TAC: total antioxidant capacity; VEGF: vascular endothelial growth factor.

Statistical study is achieved using one-way ANOVA with Tukey’s post hoc test, SPSS computer program.

* Significant difference vs. control group (*P* < 0.05).

# Significant difference vs. diabetic group without retinopathy (*P* < 0.05).

▴Significant difference vs. nonproliferative diabetic retinopathy group (*P* < 0.05).

◊ Significant difference vs. proliferative diabetic retinopathy group (*P* < 0.05).

**Figure 1.**
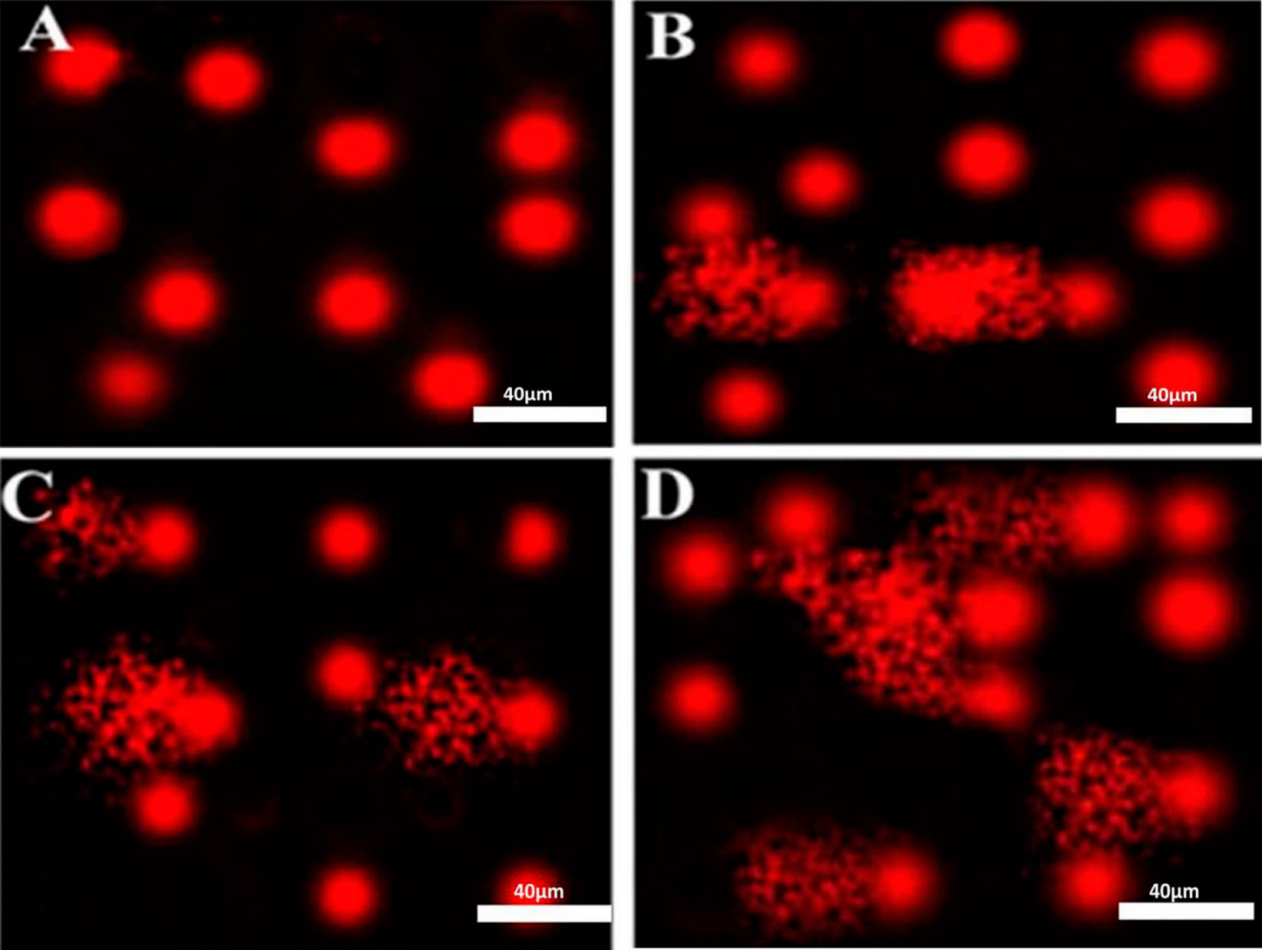
Photomicrographs representation of DNA damage in peripheral blood mononuclear cells using comet assay in control group (A), diabetic group without retinopathy (B), nonproliferative diabetic retinopathy group (C), proliferative diabetic retinopathy group (D). (The scale bar is 40 µm, 5 fields were assessed to provide this representative image).

Similarly, a significant increase in serum level of NO and peroxynitrite in diabetics was also revealed. The results of comet assay were presented in Table 3 and Figure 1. Higher significant DNA damage was recognized in diabetic groups with more obvious DNA damage in PDR group as indicated by significant increase in tail DNA%, tail length, and tail moment as compared to control healthy group.

**Table 3.** Comet assay parameters obtained by image analysis in peripheral blood mononuclear cells of the studied groups.

Variable	Control	Diabetic without retinopathy	Nonproliferative diabetic retinopathy	Proliferative diabetic retinopathy	*P* value
Tails length (µm)	1.36 ± 0.06	2.35 ± 0.13*^▴◊^	4.58 ± 0.42*^# ◊^	9.75 ± 0.37*^#▴^	**<0**.001*
Tail DNA%	1.46 ± 0.04	2.69 ± 0.06*^▴◊^	3.67± 0.08*^# ◊^	8.66 ± 1.01*^#▴^	**<0**.001*
Tail moment	1.99± 0.08	6.32 ± 0.21*^▴◊^	16.81 ± 1.45*^# ◊^	84.60 ± 6.75*^#▴^	**<0**.001*

Note: Statistical study is achieved using one-way ANOVA with Tukey’s post hoc test, SPSS computer program.

* Significant difference vs. control group (*P* < 0.05).

# Significant difference vs. diabetic group without retinopathy (*P* < 0.05).

▴Significant difference vs. nonproliferative diabetic retinopathy group (*P* < 0.05).

◊ Significant difference vs. proliferative diabetic retinopathy group (*P* < 0.05).

### 3.3. Comparisons of HIF1A antisense RNA 2 (HIF1A–AS2) relative expression between studied groups

**Figure 2.**
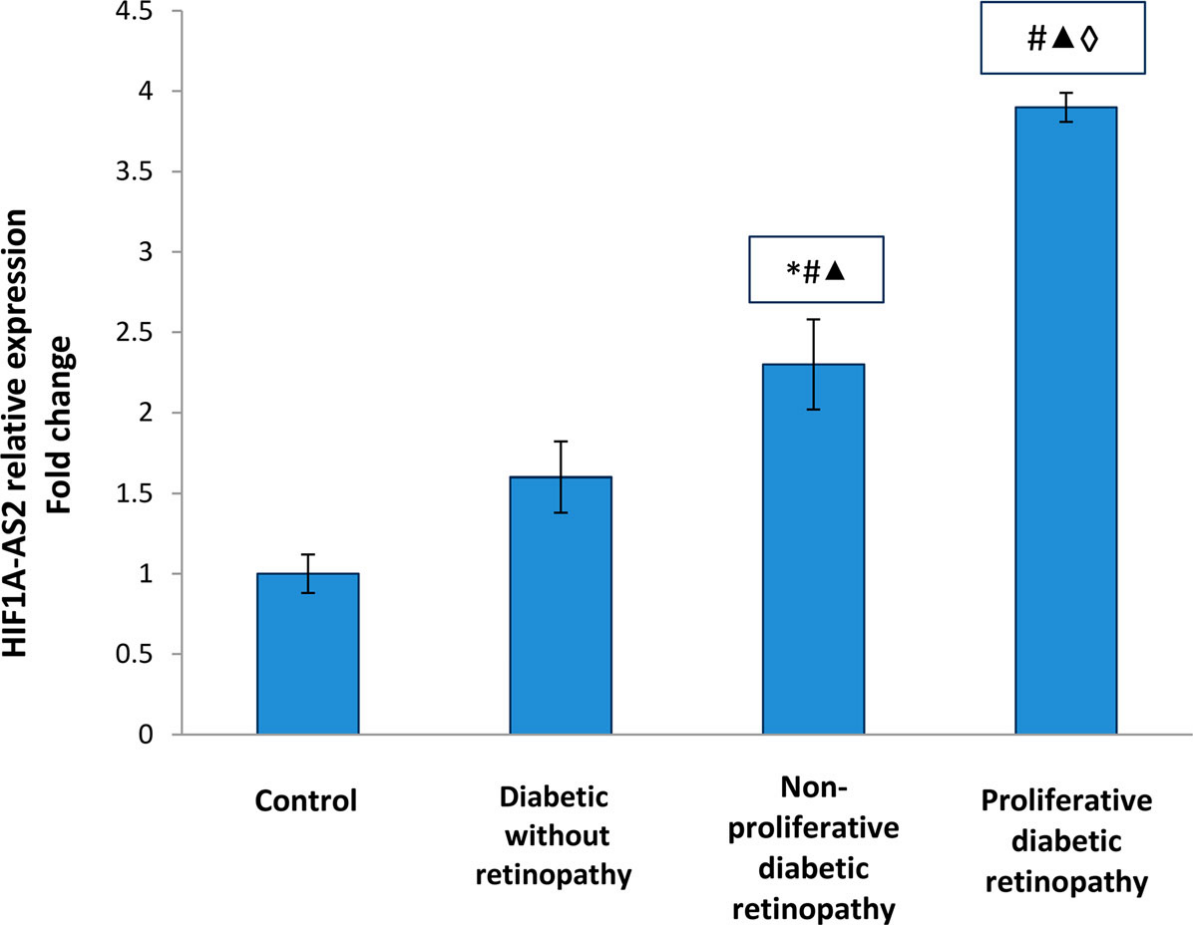
HIF1A–AS2 relative expression in the studied groups. Data are represented as mean ± SD. * Significant difference vs. control group (*P* < 0.05). # Significant difference vs. diabetic group without retinopathy (*P* < 0.05). ▴Significant difference vs. nonproliferative diabetic retinopathy group (*P* < 0.05). ◊ Significant difference vs. proliferative diabetic retinopathy group (*P* < 0.05).

As demonstrated in Figure 2, the present study revealed significant up-regulation in HIF1A–AS2 relative expressions in diabetic patients groups compared to the control group (*p* <0.001) with higher up-regulation detected in PDR group.

### 3.4. Correlations between the studied parameters

As presented in Table 4, using Pearson’s correlation, the correlations between the studied parameters were performed, displaying a significant positive correlation between HIF1A–AS2 gene expressions and HIF-1α, VEGF, MAPK, Endoglin, AGEs, MDA, NO, peroxynitrite levels, tail DNA%, tail moment, and HbA1c. However, a significant negative correlation was revealed between HIF1A–AS2 gene expressions and TAC and total thiols levels.

**Table 4.** Correlations between the studied parameters in diabetic retinopathy patients.

Variable		HIF1A–AS2	HIF-1α	VEGF	MAPK	Endoglin	MAPK	AGEs
HIF1A–AS2	**r**							
	***P***							
HIF-1α	**r**	0.719						
	***P***	<0.001*						
VEGF	**r**	0.487	0.613					
	***P***	<0.001*	<0.001*					
MAPK	**r**	0.460	0.644	0.813				
	***P***	<0.001*	<0.001*	<0.001*				
Endoglin	**r**	0.713	0.901	0.669	0.665			
	***P***	<0.001*	<0.001*	<0.001*	<0.001*			
AGEs	**r**	0.888	0.902	0.821	0.835	0.893		
	***P***	<0.001*	<0.001*	<0.001*	<0.001*	<0.001*		
MDA	**r**	0.578	0.800	0.834	0.829	0.785	0.829	0.871
	***P***	<0.001*	<0.001*	<0.001*	<0.001*	<0.001*	<0.001*	<0.001*
TAC	**r**	0.264-	0.335-	0.626-	0.641-	0.477-	0.641-	0.783-
	***P***	<0.05*	<0.001*	<0.001*	<0.001*	<0.001*	<0.001*	<0.001*
Total thiols	**r**	0.448-	0.619-	0.827-	0.885-	0.675-	0.885-	0.863-
	***P***	<0.001*	<0.001*	<0.001*	<0.001*	<0.001*	<0.001*	<0.001*
NO	**r**	0.459	0.672	0.770	0.828	0.710	0.828	0.841
	***P***	<0.001*	<0.001*	<0.001*	<0.001*	<0.001*	<0.001*	<0.001*
ONOO^−^	**r**	0.416	0.607	0.825	0.804	0.628	0.804	0.840
	***P***	<0.001*	<0.001*	<0.001*	<0.001*	<0.001*	<0.001*	<0.001*
Tail DNA%	**r**	0.740	0.936	0.568	0.674	0.837	0.674	0.848
	***P***	<0.001*	<0.001*	<0.001*	<0.001*	<0.001*	<0.001*	<0.001*
Tail moment	**r**	0.746	0.931	0.839-	0.648	0.815	0.648	0.779
	***P***	<0.001*	<0.001*	<0.001*	<0.001*	<0.001*	<0.001*	<0.001*
HbA1c %	**r**	0.470	0.673	0.395	0.401	0.624	0.401	0.859
	***P***	<0.001*	<0.001*	<0.001*	<0.001*	<0.001*	<0.001*	<0.001*

Abbreviation: AGEs: advanced glycation end products; HbA1c: glycated hemoglobin; HIF-1α: hypoxia inducible factor 1-alpha; HIF1A–AS2: HIF1A antisense RNA 2; MAPK, Mitogen activated protein kinase; MDA, Malondialdehyde; NO, nitric oxide; ONOO−, Peroxynitrite; TAC, Total antioxidant capacity; VEGF, Vascular endothelial growth factor. *P* < 0.05 is significant. *r*, Pearson’s correlation coefficient.

### 3.5. Multiple linear regression analysis of HIF1A–AS2-related factors

Multiple linear regression analysis was performed using HbA1c as dependent variable, with the other studied parameters as independent variables; the results yield that HIF1A–AS2 relative expression (B 0.460, *P* < 0.001*) was the independent predictor for PDR as shown in Table 5.

**Table 5.** Multiple linear regression analysis for potential predictors of proliferative diabetic retinopathy.

	Unstandardized coefficients		Standardized coefficients		
Variable	B	Standard error	Beta	t	*P* value
HIF1A–AS2	0.460	0.161	0.407	2.863	<0.001*
HIF-1α	0.001-	0.002	0.139-	0.614-	0.542
VEGF	0.002-	0.003	0.074-	0.530-	0.599
MAPK	0.025-	0.015	0.309-	1.927-	0.090
Endoglin	0.404	0.207	0.338	1.992	<0.05 *
AGEs	0.020	0.010	0.343	1.997	<0.05 *
MDA	0.014-	0.009	0.319-	1.562-	0.125
TAC	0.587-	0.754	0.110-	0.779-	0.440
Total thiols	0.014-	0.010	0.281-	1.323-	0.792
NO	0.020	0.015	0.161	1.336	0.188
ONOO^−^	0.255	0.140	0.263	1.816	0.076
Dependent variable: HbA1c %					

Abbreviation: AGEs: advanced glycation end products; HbA1c: glycated hemoglobin; HIF-1α: hypoxia inducible factor 1-alpha; HIF1A–AS2: HIF1A antisense RNA 2; MAPK: mitogen activated protein kinase; MDA: malondialdehyde; NO: nitric oxide; ONOO−: peroxynitrite; TAC: total antioxidant capacity; VEGF: vascular endothelial growth factor. *P* < 0.05 is significant. *r*: Pearson’s correlation coefficient.

#### 3.5.1. ROC curve of HIF1A–AS2 relative expression for discriminating NPDR and PDR from healthy controls (*available in supplementary data)*

ROC curve was applied to measure HIF1A–AS2 relative expression value as an early marker for NPDR (Figure 3A, see Supplementary material) and PDR (Figure 3B, see Supplementary material). The optimal cutoff point for NPDR group was 2.02 with sensitivity 89% and specificity 92% with an AUC 0.937, while the optimal cutoff point in case of PDR group was 2.96 with sensitivity 96% and specificity 93% with an AUC 0.972.

## 4. Discussion

Diabetic retinopathy (DR) represents a communal complication of DM and is considered to be a progressively conjoint cause of visual impairment. Blood vessel damage arises with disease advancement, with subsequent ischemia, neovascularization, blood–retina barrier (BRB) disruption, and subsequent blindness. Though research work and treatment strategies have been established noticeably over the former years, there is a scope for an improved understanding of the pathophysiology and biochemical alterations concerning the disease [34].

Currently, scientists have focused their attention on the imminent role of lncRNAs as newly revealed key players involved in the development of numerous human diseases [35]. LncRNAs, as a class of nonprotein coding RNAs, were elaborated in modulating the protein-coding genes expression at all the regulation levels, comprising transcriptional, post-transcriptional, translational, post-translation control as well as epigenetic regulation [36].

It is appealing to speculate that LncRNAs are up-regulated upon hypoxia, acting directly or indirectly to stimulate or inhibit HIF-pathway [37]. It was also established that they may be involved in regulating both pathophysiologic processes and angiogenesis during ischemic stroke since their expression profiles were altered in stroke patient’s peripheral blood [38].

LncRNA HIF1A antisense RNA 2 (HIF1A-AS2) was proven to control HIF-1α mRNA expression because the known HIF-1 protein binding sites were expected to be situated in the HIF1A-AS2 promoter area [39]. Additionally, it was described that LncRNA HIF1A-AS2 might stimulate angiogenesis in human umbilical vein endothelial cells during hypoxia by allowing HIF-1 α up-regulation. Moreover, it accelerates HIF-1α up-regulation by acting as a ‘sponge’ to miR-153-3p, which diminished the HIF-1α post-transcriptional silencing [39].

HIF1A-AS2 was categorized in hypoxia-related carcinogenesis processes, which enables adaptive cancer cells survival during hypoxic conditions. Likewise, it was interrelated to insulin-like growth factor-2 mRNA-binding protein 2 and ATP-dependent RNA helicase A, whereby endorsing the self-renewal and growth of mesenchymal glioblastoma stem-like cells in hypoxic conditions [40].

In this context, it was stated that down-regulation of HIF1A-AS1 could lessen ventricular remodeling and recover mice cardiac functions after myocardial I/R injury [41]. Concomitant with our result herein, a former study revealed a positive correlation between lncRNA HIF1A-AS2, HIF1-α, VEGF, and ANG1 in cerebral stroke [42].

Indeed, hypoxia has been implicated as a prospective critical contributor to the pathogenesis of many retinal diseases, including DR and the cellular response toward hypoxia is transcriptionally regulated by HIF [43]. Likewise, it was thought that DR is strictly interrelated with glucose metabolism and microvascular status, particularly glucose metabolic disorder, micro-vessels alternation, and blood flow blockade. These events generate a state of microcirculation dysfunction, hypoxia as well as retinal ischemic status with subsequent retinopathy [44].

The HIFα-HIFβ complex can trigger transcription of genes with promoters presenting hypoxia response elements comprising erythropoietin and VEGF as during hypoxia; HIF-1 is expressed, binds to DNA, and tempts VEGF mRNA transcription [45]. Moreover, numerous research works supported the notion that hypoxia is a dynamic force in DR progression, rather than an outcome [46,47]. Consistent with this hypothesis, hypoxia itself has been verified to stimulate production of a wide variety of proangiogenic factors such as VEGF, one of the principal targets of therapeutic management in DR [48–50].

Similarly, studies have established that at the initial and late stages of DM, HIF-1α and VEGF together play a significant role through diverse disease stages by incorporating into glycolytic activity during hypoxia, to enable the body to adapt to its own internal environment [51]. Furthermore, in ischemic retinal tissue, another study documented up-regulated HIF-1α expression that is momentarily and space-specific increased with the augmented expression of VEGF. So, this result matches the assumption that HIF-1α has an important role in the increment of the expression of VEGF [52,53].

Similarly, prevalent independent research studies have discovered that the initial biochemical and retinal pathology alterations seem to begin within the first week of the time when the animals become diabetic. These include the formation of AGEs, the overproduction of VEGF and its mRNA, and the subsequent leak of capillary endothelial cells, which causes retinal hypoxia [54,55]. From another perspective, it was reported that HIF-1α phosphorylation is performed by the P42/P44/MAPK pathway in mice endothelial 1G11 cells, and this activation can trigger HIF-1α transcription, thus increasing the expression of HIF-1α-induced genes [56].

Furthermore, it was found that MAPK phosphorylation, motivated by hypoxia, is perilous to HIF-1α activity, comprising attenuating its ubiquitination and promoting its nuclear translocation, so that HIF-1α enters the nucleus to act as transcriptional factor to regulate cell survival in hypoxic conditions [57].

Notably, whether MAPK initiation controls the HIF-1-regulated cascade, which includes the activity, stability, and task of HIF-1 target genes like VEGF, or whether MAPK is a downstream goal of HIF-1 with inverse regulatory consequences, the comprehensive mechanism needs to be better demonstrated [58].

Endoglin is a cell-surface coreceptor for the TGF-β1 that is highly expressed in the endothelial. It has a crucial role in angiogenesis, endothelial dysfunction, and diabetic complications [59]. Endoglin is indispensible for normal angiogenesis, so its expression is up-regulated during healing of wounds, atherosclerosis, inflammation, hypoxia, and vascular injury as well as in developing embryos [60]. This hypoxia-related up-regulation of endoglin gene expression may be arbitrated through p38MAPK signaling pathway since its hypoxic up-regulation was abridged by p38 inhibitors and by a prevailing negative state of the p38-activating kinase [61].

It has been detected that oxidative stress (OS) is a mutual denominator link for the major pathways which are involved in the diabetes progression and complications. Elevated glucose levels prompt intracellular reactive oxygen species (ROS) either directly by glucose metabolism and auto-oxidation or indirectly by creating AGE products [62]. Naruse et al. [63] recommended that DR causes an upsurge in the liberation of reactive oxygen metabolites and its progression; in type 2 DM patients, it is concomitant with increased production of biomolecules such as NO and lipoperoxides.

Additionally, the role played by OS in DR is supported by the clarifications that antioxidants conquer hyperglycemia-induced augmented liberation of mitochondrial superoxide and heightened peroxynitrite levels in the retinal capillary cells which inhibit mitochondrial dysfunction and cellular apoptosis in experimental diabetic animal’s retinal cells [64,65]. Moreover, extreme quantities of ROS oxidize bio-molecules such as protein and DNA, following beating various anti-oxidative defense mechanisms, which leads to OS development [66].

A comet assay is believed to be a subtle method for defining DNA strand breaks and oxidative DNA base damage at the level of the cell [67]. In the current research study, an escalation in tail length, tail DNA%, and tail moment was observed representing severe OS that eventually led to oxidative DNA damage. This came in agreement with our results herein as we documented the existence of significant surge in oxidative DNA damage in diseased groups with the highest level in group III.

## 5. Conclusion

In conclusion, given the importance of lncRNAs in better understanding disease pathogenesis and evolution, the insight gained from this study provides necessary understanding for the collaboration between LncRNA/HIF1A antisense RNA 2, HIF-1, VEGF, and Endogolin, as well as redox status and comet assay, which are profoundly subsidizing to the pathogenesis of DR and linked to its severity by monitoring their levels at different stages.

These indicators are eligible to be unique noninvasive biomarker panels for DR monitoring, as evidenced by their substantial connection with DR severity. As a result, the available data might be used in clinical trials to assist future treatment methods targeted at treating DR and avoiding further deterioration.

## 6. Limitations

For better data validation, the study could be performed on a larger sample of population and could be supported experimentally in the upcoming research.

## Supplementary Material

Supplemental Material

## Data Availability

Research data are not publically shared.
