# Supplementary material for: The evolving role of long noncoding RNA HIF1A-AS2 in diabetic retinopathy: a cross-link axis between hypoxia, oxidative stress and angiogenesis via MAPK/VEGF-dependent pathway
Source: Redox Rep. 2022 Mar 14;27(1):70–8. doi: 10.1080/13510002.2022.2050086 (PMC8928809; doi:10.1080/13510002.2022.2050086)
Supplement: Supplemental Material [file YRER_A_2050086_SM4124.docx]

***Original Article***

**Title:**

**The Evolving Role of Long Non-coding RNA HIF1A-AS2 in Diabetic Retinopathy: A Cross-link Axis between Hypoxia, Oxidative Stress and Angiogenesis via MAPK/VEGF Dependent Pathway**

**Marwa Mohamed Atef ^1^, Noha M. Shafik^*1^, Yasser Mostafa Hafez ^2^,** **Mona Mohamed Watany ^3^, Amal Selim ^2^,** [**Heba M. Shafik**](https://www.researchgate.net/profile/Heba-Shafik-2) **^4^, Omnia Safwat El-Deeb^1^**

^1^ Medical Biochemistry Department, Faculty of Medicine, Tanta University, Tanta, Egypt

^2^ Internal Medicine Department, Faculty of Medicine, Tanta University, Tanta, Egypt

^3^ Clinical pathology Department, Faculty of Medicine, Tanta University, Tanta, Egypt

^4^ Ophthalmology Department, Faculty of Medicine, Tanta University, Tanta, Egypt

***Corresponding author:**

**Noha M. Shafik**

Assistant Professor of Medical Biochemistry,

Address: Medical Biochemistry Department, Faculty of Medicine, Tanta University, El Geesh street, Tanta, Egypt

E-mail:

[noha.said@med.tanta.edu.eg](mailto:noha.said@med.tanta.edu.eg)

nohashafik2008@yahoo.com

ORCID ID: http://orcid.org/0000-0003-3191-8300

Postal code: 31511


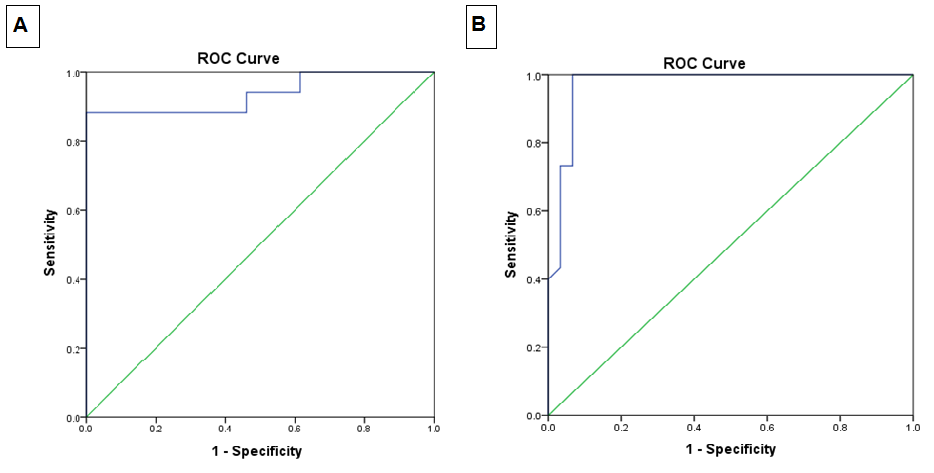


**Figure 3:** ROC curve of HIF1A‑AS2 relative expression for discriminating

A: Non-proliferative diabetic retinopathy (NPDR) patients from healthy controls;

B: Proliferative diabetic retinopathy (PDR) patients from healthy control.

**Results:**

**ROC curve of HIF1A‑AS2 relative expression for discriminating NPDR and PDR from healthy controls**

ROC curve was applied to measure HIF1A‑AS2 relative expression value as an early marker for NPDR (figure 3A) and PDR (figure 3B). The optimal cut-off point for NPDR group was 2.02 with sensitivity 89% and specificity 92% with an AUC 0.937. while the optimal cut-off point in case of PDR group was 2.96 with sensitivity 96% and specificity 93% with an AUC 0.972.
